# New ways of treatment of fractures of the humeral shaft: does the combination of intramedullary nail osteosynthesis and cerclage improve the healing process?

**DOI:** 10.1007/s00068-021-01847-1

**Published:** 2021-12-31

**Authors:** Franziska von der Helm, Annabel Fenwick, Jan Reuter, Leonard Adolf-Lisitano, Edgar Mayr, Stefan Förch

**Affiliations:** Klinik für Unfallchirurgie, Orthopädie, Plastische und Handchirurgie, University Hospital of Augsburg, Augsburg, Germany

**Keywords:** Humeral shaft fracture, Cerclage, Antegrade intramedullary nail osteosynthesis of the humerus, Lesion of the radial nerve, Non-union

## Abstract

**Introduction:**

The humeral shaft fracture is a rare fracture of the long bones with various treatment options. Dreaded complications such as lesions of the radial nerve or non-unions make the decision for what kind of therapy option more difficult. Biomechanically the upper arm is mostly exposed to rotational forces, which affect intramedullary nail osteosynthesis. Additive cerclage may compensate for these in spiral fractures. The aim of this study is to investigate what effect a combination of intramedullary nail osteosynthesis and limited invasive cerclage has on the rate of healing. In addition, this study addresses the question if complications arise as a result of cerclage.

**Methods:**

In this retrospective study, 109 patients were evaluated, who, during a period of 6 years, underwent operative treatment of a humerus shaft fracture with a combination of intramedullary nail osteosynthesis and additive cerclage. The primary end point was to establish the rate of healing. A secondary end point was to evaluate complications such as infections and damage to the nerve. This was followed by an examination of patient files and X-ray images and a statistical analysis with SPSS.

**Results and conclusion:**

The healing process shows a non-union rate of 2.6%, and complications such as secondary radial nerve lesions of 4.6%. The antegrade intramedullary nail osteosynthesis with limited invasive, additive cerclage reduces the risk of non-union and does not lead to an increased risk of iatrogenic damage to the radial nerve. Wound healing was not impaired and there were no infections through the cerclage in our patient cohort.

## Introduction

The fracture of the humeral shaft is a rare fracture with a frequency of 1–3% of all fractures and nearly 20% of all humeral fractures [1, 2]. Apart from high energy trauma such as falls from great height and traffic accidents also low energy trauma such as falls from stumbling by elderly people is a common accident mechanism [3]. The therapy of the humeral shaft fracture is manifold and controversial as a result of its anatomic relationship to the radial nerve and because of the high likeliness to develop a non-union [4, 5]. Previously it belonged to the domain of conservative treatment with immobilization in a brace according to Sarmiento et al. [6]. In the operative treatment intramedullary nailing and plate osteosynthesis are increasingly in competition with each other and allow a load free, early functional postoperative treatment [2, 7–9]. Osteoporotic fractures in elderly people often lead to delayed healing of the fracture or even to non-union and implant failure and are linked to a considerable morbidity and a longer convalescence [5]. Biomechanically the osteosynthesis at the upper extremity is above all affected by rotational forces. Intramedullary nail osteosynthesis are particularly affected by this. Additive cerclage compensate rotational forces if anatomically reduced, as could be shown in an experimental study of a tibia model with plate osteosynthesis together with additive cerclage [10]. Therefore, from a biomechanical point of view an additive cerclage especially in intramedullary nailing on the humeral shaft seems sensible.

The arguments against their use in upper arm fractures are on one hand the risk of iatrogenic damage to the radial nerve, and on the other hand, a presumed impairment of blood circulation of the fragment [11]. The aim of this retrospective study is the analysis of the healing process and complications such as radial nerve damage when additive, limited invasive cerclage in antegrade nail osteosynthesis of humeral shaft fractures are used.

## Methods

### Surgical technique

**Fig. 1:**
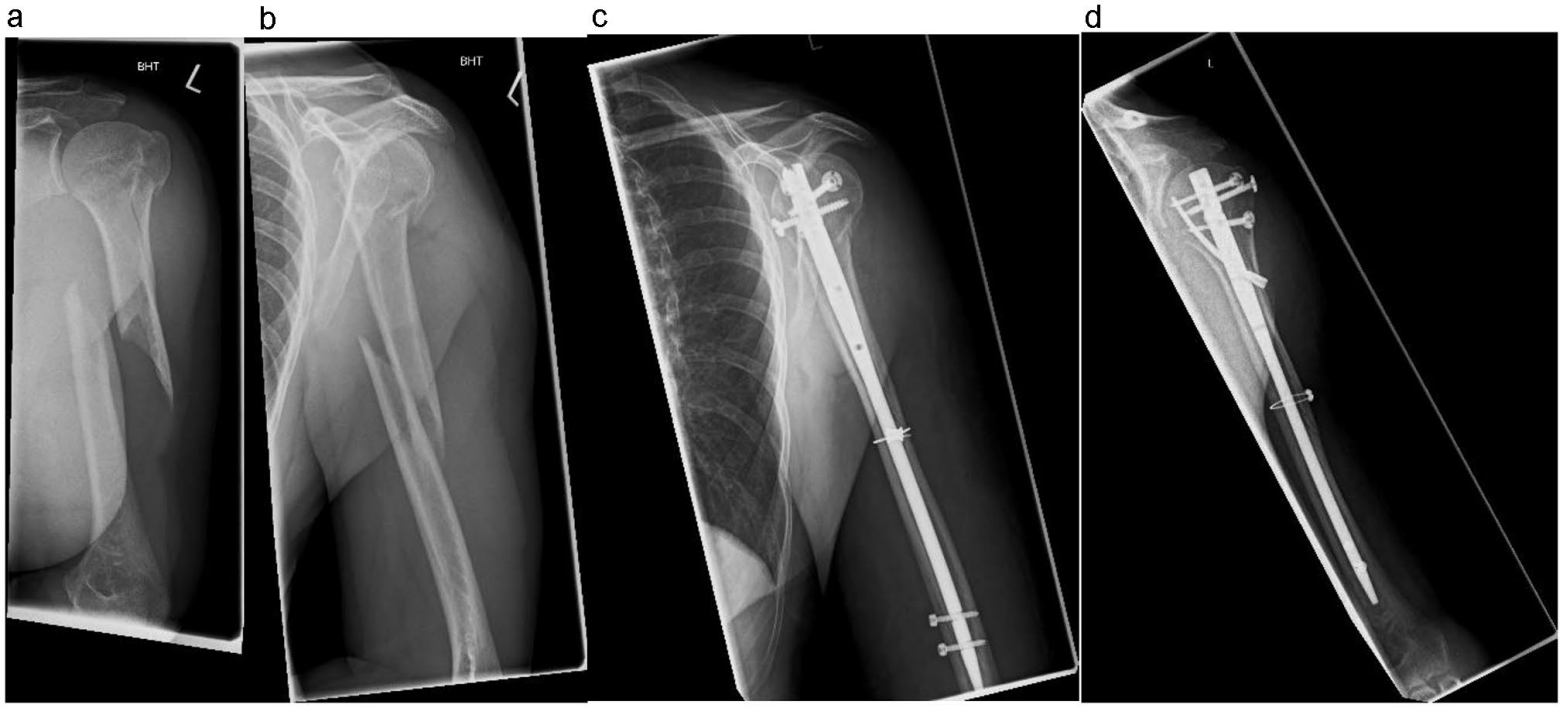
Pre- and post-operative imaging of a humeral shaft fracture AO 12 A1 left. **a**, **b** Pre-operative imaging in two planes (ap/lateral). **c**, **d** Post-operative imaging in two planes (ap/lateral)

**Fig. 2:**
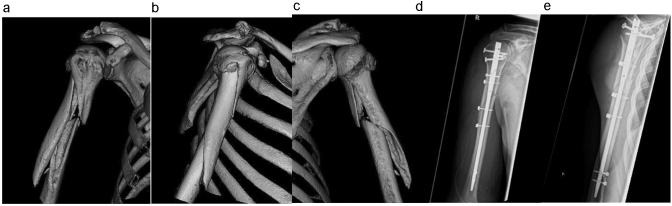
A humeral shaft fracture AO 12 B2 right. **a**–**c** Pre-operative imaging (computed tomographic 3D reconstruction) and **d**, **e** post-operative imaging in two planes (ap/lateral)

The antegrade intramedullary nail osteosynthesis (Locking Blade Nail (LBN), Marquardt Medizintechnik GmbH) with additive, limited invasive wire cerclage (DePuy Synthes, Johnson & Johnson Medical GmbH) is performed routinely in Beach-Chair-Position [12]. The height of the planned cerclage is defined radiographically and followed by an incision of 6–8 cm on the lateral side of the upper arm. After incising the fascia the muscles are carefully pushed of the humerus with a rasparatory. A rider followed by a Redon drain (B.Braun, Redon Suction Drain, U2110800, CH08) are inserted. It is grasped with an overholt clamp and retrieved on the lateral side of the wound. The cerclage (DePuy Synthes, Cable System, REF 298.801.01, ∅ 1.7 mm) is inserted into the Redon drainage and is pulled through wrapping itself around the fracture fragments of the humerus. The integrity of the radial nerve is checked manually. Axial traction and rotation of the distal fragment is used to reduce the fracture anatomically with simultaneous tightening of the cerclage. If further cerclages are required, the described procedure is repeated accordingly. Preliminary stabilization is followed by antegrade intramedullary nailing of the humerus via a delta split approach using the standard technique. Figures 1 and 2 show examples of the described surgical treatment.

For retrospective analysis, all patients were included who, within a period of 6 years (January 2015–December 2020), were treated operatively for a humeral shaft fracture as described above. Criteria for inclusion were age > 18 years, spiral and wedge fractures, which were treated operatively. Criteria for exclusion were pathological fractures, multiple injuries of the ipsilateral extremity in polytraumatized patients, horizontal fractures (< 30°) (AO 12-A3), comminuted fractures (AO 12-C3) and fractures where the distal upper arm was also affected as these are not suitable for stabilization with additive cerclage. Via the hospital data system, the following data were established: age, gender, ASA score, accompanying illnesses and length of hospital stay. The secondary diagnosis of osteoporosis was recorded as part of the geriatric assessment. The accident mechanism was analysed. High-energy traumas are injuries that affect the body surface with high kinetic energy such as traffic accidents and falls from great heights. Low energy trauma was defined as a fall from a standing position or from a low height of less than 1 m. From the operation reports, the intraoperative and postoperative complications as well as blood loss were recorded in addition to the side, duration of the operation and soft tissue damage.

With the help of the preoperative radiographs the fracture morphology according to AO/OTA was classified [13]. The number of cerclages was determined preoperatively after analysis of the fracture morphology and intraoperatively by the respective surgeon. The aim is to achieve anatomical reduction and sufficient rotational stability with as few cerclages as possible.

All complications were recorded and evaluated. Each patient with a complicative course of treatment was contacted and re-examined. In case of a primary or secondary damage to the radial nerve, whilst still in postoperative primary hospital care, a neurological examination including neuro sonography and measuring of the nerve conduction velocity was performed. Necessary further revision surgeries were recorded as well as late residues after diagnosed injury of the radial nerve.

To document the healing process, each patient was contacted. Correct bony healing was only counted if a radiograph was available and the patient was symptom-free.

The analysis and graphical representation was carried out using IBM SPSS Statistics version 27.0 (SPSS Inc., Chicago, IL, USA).

A positive ethics committee vote (file no. 2019-36) was acquired.

## Results

Altogether 109 patients over a period of 6 years (January 2015–December 2020) were included and underwent a follow-up examination in this study. The cohort consisted of 45 male and 64 female patients. The average age of female patients was 74 years (37–95) and thus higher than that of male patients with 64 years (28–89). Similarly, there were more secondary illnesses such as osteoporosis and renal dysfunction in the female patient group. Alcohol consumption was higher amongst male patients (Tables 1, 2).

**Table 1:** Co-morbidities of the investigated patients

Gender	Men (*n* = 45) (%)	Female (*n* = 64) (%)	Total (*n* = 109) (%)
Osteoporosis	28.9	68.8	52.3
Diabetes	13.3	14.1	13.8
Renal dysfunction	37.8	78.1	61.5
Adipositas	20	14.1	16.5
Alcohol abuse	53.3	7.8	26.6
Smoking	48.9	10.9	25.7
Direct oral anticoagulants	37.8	39.1	39.5
Multi medication	28.9	58.8	49.5

**Table 2:** Patient condition pre-operatively according to ASA (American Society of Anesthesiologists)

ASA 1	Healthy patient	8.3%
ASA 2	Patient with mild diseases	43.1%
ASA 3	Patient with severe diseases	45.9%
ASA 4	Servere disease life threatening	3%

In 23 cases (21.1%), a high energy trauma and in 86 cases (78.9%) a low energy trauma was the cause of accident. In 101 patients this was an isolated injury, whereas in 8 patients the injury occurred within a polytrauma (Table 3). In 55 cases the fracture was localized on the left side and in 54 cases on the right hand side.

**Table 3:** Accident mechanisms

High energy trauma (*n* = 23)		Low energy trauma (*n* = 86)
Traffic accidents (4/23)	17.4%	Domestic falls 61.6% (53/86) (Under alcohol 8/53)
Fall from bicycle/sports (8/23)	34.8%	
Fall from building scaffolding (5/23)	21.7%	Fall in the street 38.4% (33/86) (Under alcohol 17/33)
Fall on stairs (6/23)	26.1%	

**Fig. 3:**
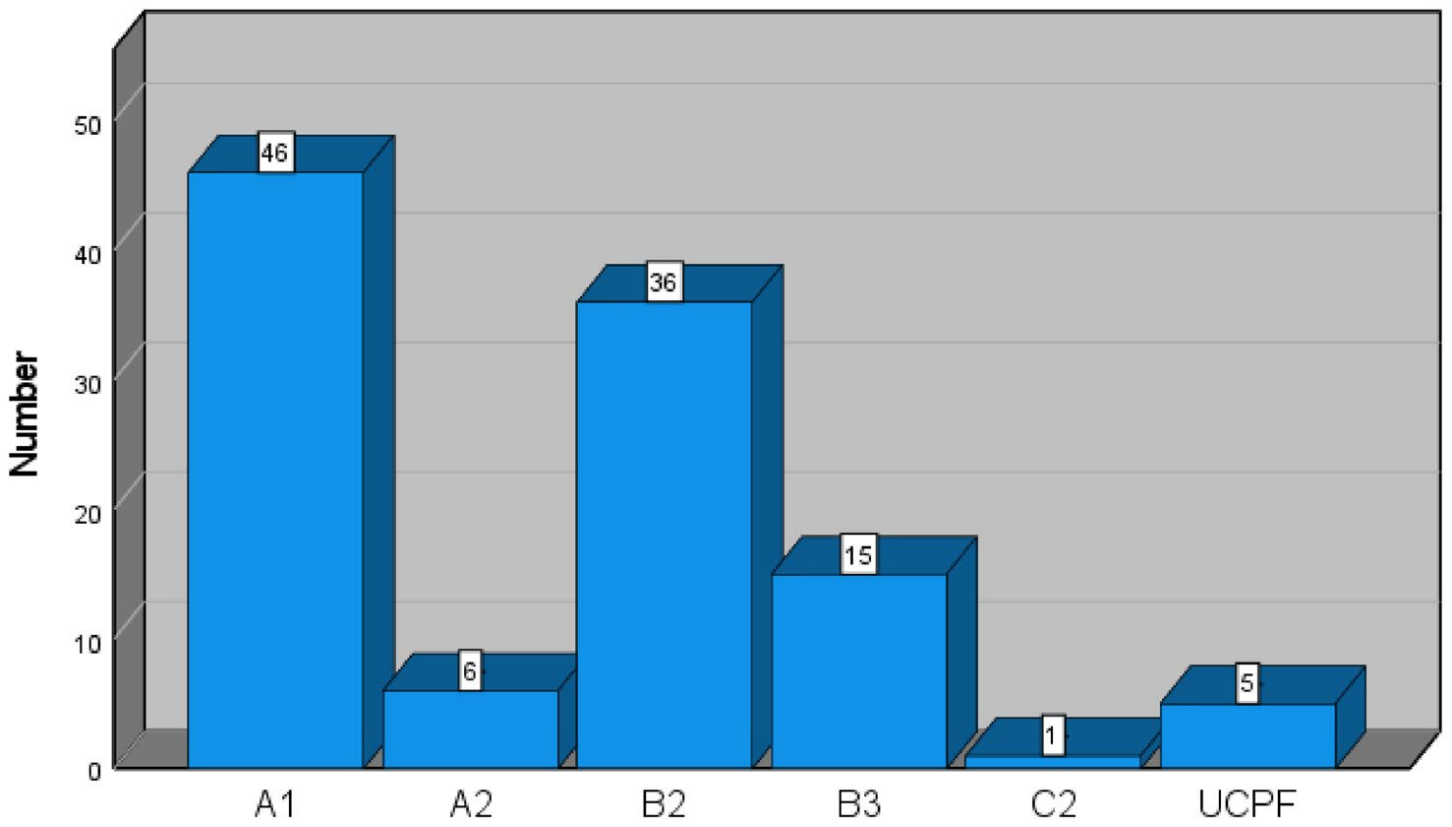
Distribution of fractures by AO classification; periosteosynthetic fracture [unified classification system for periprosthetic fractures (UCPF)]

The evaluation of the pre-operative radiological images shows, in line with the fracture classification according to AO/OTA, fractures of type 12-A in 52 patients, of type 12-B in 51 patients and of type 12-C in 1 patient. Five patients had a periosteosynthetic fracture (Fig. 3).

The operation took place on average 1.62 days after hospital admission. Seventy-six operations took place within 24 h. The average time of surgical procedure was 115 min (51–204 min) and the intraoperative loss of blood averaged 265 ml (50–700 ml). Here there was no difference between the patients on antiplatelet medication like aspirin (ASS), direct oral anticoagulants (DOACs) or warfarin. The average hospital length of stay was 9 days for mono injuries and 15 days (2–36) for polytraumatized patients. Altogether 195 additive wire cerclage were used around the humerus (Table 4).

**Table 4:** Number of used cerclage

	Number of cerclages per patient	Number of operations
Cerclages (DePuy Synthes, Johnson & Johnson Medical GmbH)	1	32
	2	59
	3	17
	4	1

**Fig. 4:**
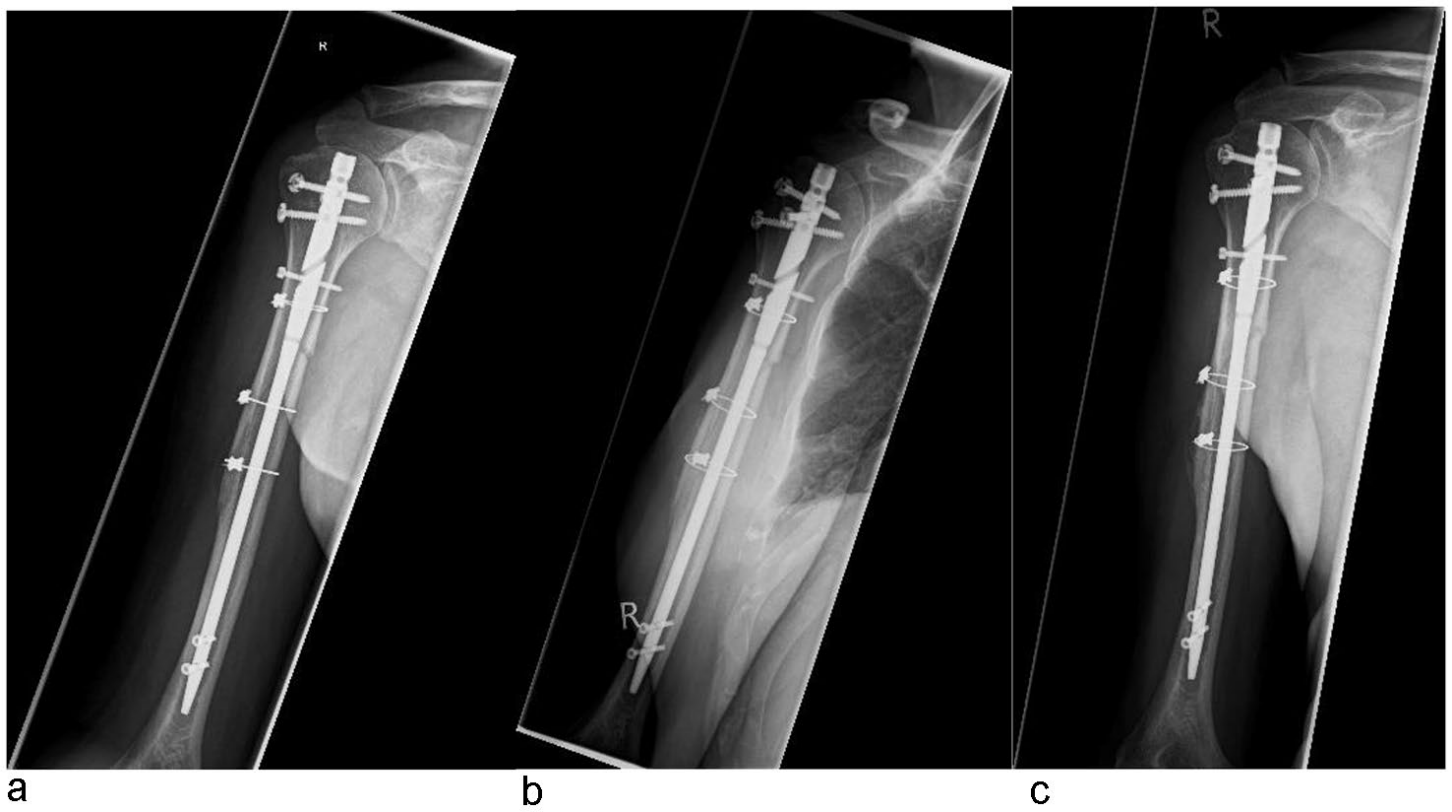
Healing process of the B2 fracture from Fig. 2. **a** The fracture gap is still clearly visible in the radiological X-ray check 6 weeks after surgical treatment. **b** Callus formation begins 12 weeks after operation. **c** Healing 6 months after the operation

The healing process in 78 patients could be documented with a radiograph (Table 5, Fig. 4). After 3–12 months, 75 patients showed a complete bony consolidation. Two patients developed a non-union. In one case, no revision surgery was carried out on the grounds auf age, low expectation and underlying medical pre-conditions. In another case the implant was removed including the cerclage, followed by filling of the defect with spongiosa from the iliac crest and plate osteosynthesis (3.5 LCP).

**Table 5:** Healing process with radiologically documented bony healing

Bony healing	Total
Total	75
3 months	18
6 months	13
9 months	5
12 months	39

In one case, revision of a hematoma became necessary. This, however, was not in the region of the cerclage, but was linked to an overdose of warfarin. One patient showed a complicated course. Initially, a secondary damage to the radial nerve manifested itself. Neurophysiological damage from the cerclage could be excluded. Subsequently, the patient suffered a periosteosynthetic fracture distally to the inlying nail after a further fall, which was treated with a double plate osteosynthesis. This led to a postoperative infection, which made several revision surgeries and a Spacer-implantation necessary.

There were 16 cases of a radial nerve damage, of which 11 lesions already existed preoperatively, that is, due to trauma. Intraoperatively, the nerve was visualised and found to be intact. In five cases (4.6%), the damage only developed postoperatively.

Neurophysiologial and neuro ultrasound examination could exclude in all cases direct damage through the cerclage and indicated traction damage. Further four revision surgeries were carried out if symptoms persisted to display the nerve. Here, it became evident that there was no macroscopic damage to the nerve intraoperatively in any case. In one case, severe tissue scaring had compressed the nerve, the intraoperative neuro stimulation carried out achieved a good motor response (Table 6).

**Table 6:** Overview of post-operative complications

Complication	Total
Non-union	2
Infection	1
Haematoma	1
Primary radial nerve damage	11
Secondary radial nerve damage	5

## Discussion

In the current literature, there is no specific algorithm for a decision on treatment of humeral shaft fractures, as there are many factors that must be considered for deciding. This is made even more difficult by the comparatively low case number. It is nevertheless important to consider of a standard approach.

One of the important points for discussion about an optimal decision for treatment is the improvement of non-union rates and the minimization of complications like damage to the radial nerve, as these situations lead to functional failures after a long course of treatment. As already mentioned in the introduction, the treatment of humeral shaft fractures was for a long time the domain of conservative therapy. With the development of modern implants and surgical procedures, but also due to changing patient demands, surgical treatment is becoming increasingly important. To enable early functional treatment, we also perform osteosynthesis for simple shaft fractures that are in principle also suitable for conservative therapy, if this corresponds to the patient’s wishes and requirements.

In our study, we could document the progress of healing in 78 patients (71.6%). Here we saw two cases of non-union (2.6%). Literature generally reports a non-union rate between 8 and 20% of the humeral shaft [14–17]. Blum et al. in their study of follow-up-examinations of humeral shaft fractures report with a similar number of cases (*n* − 75) show 12 cases of non-union after operative treatment with retrograde nail osteosynthesis (UHN) [18]. In a comparative study of antegrade nailing (*n* = 25) and plate osteosynthesis (*n* = 25) Wali et al. described two cases of non-union in each group [19].

Factors which favor non-union are, besides a high body-mass-index, smoking, alcoholism, osteoporosis, renal dysfunction and multiple medication, also fracture morphology and operative treatment [20, 21]. An experimental study by rats was able to show that episodic consumption of alcohol negatively influences the bio mechanical properties of callus [22]. The patient cohort, as summarized in Tables 1 and 2, shows many of the mentioned risk factors, which can favor non-union, but the rate of non-union is nevertheless in the lower margin of the data provided by the literature. The operative stabilization through additive cerclage has thus a positive effect on the healing process without risking the strangulation of the fragment blood circulation [11].

The perioperative management to avoid complicative courses therefore influences the therapy decision-making and the rate of the healing process. The average time of surgical procedure of 115 min for nail osteosynthesis with additive cerclage is no longer in comparison to the average time of surgery for plate osteosynthesis or singular nail osteosynthesis [23, 24]. Also the intraoperative blood loss irrespective of anticoagulants is no higher [8, 19].

An operative hematoma revision resulted from Warfarin medication, but it was not found in the access area to the cerclage. An iatrogenic lesion of the radial nerve was observed in five cases (4.6%) in this study and is therefore lower than the secondary damage to nerves described in the literature [7, 25]. The high spontaneous complete restoration of the nerve function correlates here with the data provided by the literature [25] and is a further indication that a careful use of limited invasive additive cerclage does not compromise the nerve.

In the literature, there is no consensus of the best surgical technique. The advantages of plate osteosynthesis with anatomic reposition, exposure of the nerve and direct interfragmentary compression are offset by the advantages of nail osteosyntheses, such as minor damage to the soft tissue and better aesthetical scaring as well as a shorter time of surgery. The comparison of postoperative infections, secondary paresis of the radial nerve, implant failure, delayed union and non-union have shown no significant differences in studies so far [3, 8, 9, 19]. Against this, one can presume that the surgical procedure described here, with a rate of 2.6% of fracture healing impairment without an increased rate of radial nerve damage, is superior compared to the surgical procedures without additive cerclage. But at the same time in no case could a connection be found between radial nerve damage and cerclage.

There are limitations to this study. As the data were collected retrospectively, there is no standardized radiological protocol. Therefore, only the observation on bony healing can be made, but none on the time frame until bony healing was achieved. In 96% of patients, a bony consolidation could be radiologically documented in the follow-ups.

A comparative group treated by different methods would be desirable. In our clinic, however, all humerus shaft fractures suitable for treatment with cerclage, were treated this way. The only exceptions are fractures classified by AO as 12-A3 and C3. For this reason, no comparative group could be formed from the clinic’s internal data. On the basis of similar case numbers in other studies, a comparison with the literature seems legitimate. These demonstrate that treatment with additive cerclage has lower complication rates than other surgical procedures.

In the primary end point, 21 patients (19.3%) were lost to follow-ups. But even so there is no reason to assume a complicative course. The operative treatment with LBN is not widespread regionally, and as the only trauma center Level I within a wide area, revisions would presumably take place here. The secondary final point also shows a comparatively low number of infections and damage to the nerve. In an earlier study, we could already prove that fracture treatment with cerclage does not lead to direct damage of the radial nerve. There is far more indication of damage to the nerve by intraoperative traction.

When considering all factors, the treatment of humerus shaft fractures with intramedullary nail ostesynthesis and additive cerclage is a valuable surgical method, which, after careful analysis of fracture morphology, is highly successful.
